# Gingival neoplasms: a multicenter collaborative study of 888 patients in Brazil

**DOI:** 10.4317/medoral.25707

**Published:** 2023-06-18

**Authors:** Éder Gerardo Santos-Leite, Brendo Vinicius Rodrigues Louredo, Lucas Lacerda de Souza, Helder Antônio Rebelo Pontes, Flávia Sirotheau Correa Pontes, Jean Nunes dos Santos, Águida Cristina Gomes Henriques, Jurema Freire Lisboa de Castro, Elaine Judite de Amorim Carvalho, Augusto César Leal da Silva Leonel, Raylane Farias de Albuquerque, Thayanne Oliveira de Freitas Gonçalves, Aline Corrêa Abrahão, Michelle Agostini, Mario José Romañach, Vinicius Coelho Carrard, Manoela Domingues Martins, Teresa Cristina Ribeiro Bartholomeu dos Santos, Fábio Ramôa Pires, Alan Roger Santos-Silva, Márcio Ajudarte Lopes, Pablo Agustin Vargas, Danyel Elias da Cruz Perez

**Affiliations:** 1 Oral Diagnosis Department, Piracicaba Dental School, University of Campinas, Piracicaba, São Paulo, Brazil; 2 Oral Pathology Unit, João de Barros Barreto University Hospital, Federal University of Pará, Belém, Pará, Brazil; 3 Oral Pathology Unit, School of Dentistry, Federal University of Bahia, Salvador, Bahia, Brazil; 4 Oral Pathology Unit, School of Dentistry, Universidade Federal de Pernambuco, Recife, Pernambuco, Brazil; 5 Oral Pathology Department, School of Dentistry, Federal University of Rio de Janeiro, Rio de Janeiro, Rio de Janeiro, Brazil; 6 Oral Pathology Department, School of Dentistry, Federal University of Rio Grande do Sul, Porto Alegre, Rio Grande do Sul, Brazil; 7 Oral Pathology Department, School of Dentistry, State University of Rio de Janeiro, Rio de Janeiro, Rio de Janeiro, Brazil

## Abstract

**Background:**

To evaluate the prevalence and clinicopathological features of a large series of gingival neoplasms in Brazil.

**Material and Methods:**

All gingival benign and malignant neoplasms were retrieved from the records of six Oral Pathology Services in Brazil, during a 41-year period. Clinical and demographic data, clinical diagnosis, and histopathological data were collected from the patients' clinical charts. For statistical analysis, the chi-square, median test of independent samples and the U Mann-Whitney tests were used, considering a significance of 5%.

**Results:**

From 100,026 oral lesions, 888 (0.9%) were gingival neoplasms. There were 496 (55.9%) males, with a mean age of 54.2 years. Most cases (70.3%) were malignant neoplasms. Nodules (46.2%) and ulcers (38.9%) were the most common clinical appearance for benign and malignant neoplasms, respectively. Squamous cell carcinoma (55.6%) was the most common gingival neoplasm, followed by squamous cell papilloma (19.6%). In 69 (11.1%) malignant neoplasms, the lesions were clinically considered to be inflammatory or of infectious origin. Malignant neoplasms were more common in older men, appeared with larger size, and with a time of complaint shorter than benign neoplasms (*p*<0.001).

**Conclusions:**

Benign and malignant tumors may appear as nodules in gingival tissue. In addition, malignant neoplasms, especially squamous cell carcinoma, should be considered in the differential diagnosis of persistent single gingival ulcers.

** Key words:**Gingiva, gingival neoplasms, mouth neoplasms, gingival diseases, prevalence.

## Introduction

Although most gingival diseases are of inflammatory origin, resulting from the accumulation of dental biofilm, several non-plaque-induced gingival diseases (NPIGD) may occur (1). The American Academy of Periodontics (AAP) and the European Federation of Periodontics (EFP), at the 2017 World Workshop on Periodontal and Peri-Implant Diseases and Conditions, proposed a classification for the NPIGD based on their etiology (1). Among the diseases classified as NPIGD, there are malignant neoplasms and oral manifestations of systemic diseases, which have substantial clinical significance (2,3). Other diseases classified as NPIGD are genetic/developmental abnormalities, specific bacterial, fungal, and viral infections, inflammatory and immune conditions/lesions, reactive processes, endocrine, nutritional, and metabolic diseases, traumatic lesions, and gingival pigmentation (1,2).

The 2017 World Workshop on Periodontal and Peri-Implant Diseases and Conditions presents a more comprehensive and understandable collection of NPIGD. However, the classification and distribution of gingival neoplasms still needs improvement because their high clinical significance. In the current classification, no group includes benign gingival neoplasms, nor the description of gingival metastases and sarcomas. The gingival malignant neoplasms, primary or metastatic, present unespecific clinical characteristics. They often mimic inflammatory or reactional conditions, and in early stages, they may be misdiagnosed as an inflammatory periodontal condition (1,4,5).

The squamous cell carcinoma (SCC) is the most common neoplasm of the gingiva, representing about 10 to 20% of all oral cancers (1,6). However, in addition to SCC, other neoplasms can affect the gingival tissue, such as benign neoplasms, leukemic infiltrations, lymphomas, and sarcomas (2,7,8). Gingival metastases may also occur. Previous studies have shown that about 50% of the oral soft tissue metastases occur in gingiva (9,10).

Most studies assessing the distribution and prevalence of NPIGD included diseases of different etiology, not specifically neoplastic lesions (2,11), and demonstrate that 76.3% of gingival diseases are NPIGD (2). The dentists have a key role in the early identification and diagnosis of these tumors. Studies evaluating the prevalence and clinicopathological features of gingival neoplasms may be important to warning dentists, both general practitioners and specialists, about these diseases. There are few studies evaluating the prevalence and clinicopathological characteristics of gingival neoplasms. These studies have shown a prevalence ranging from 7.6-35.9% of neoplasms among NPIGD, with malignant neoplasms being the most frequent in these populations (7,12). Most studies have evaluated only malignant tumors, particularly SCC, or represent small case series or single case reports (13,14). Thus, the objective of this interinstitutional collaborative study was to evaluate the prevalence and clinicopathological features of a large series of gingival benign and malignant neoplasms from Brazil.

## Material and Methods

This study was approved by the Research Ethics Committee of the Piracicaba Dental School, University of Campinas (UNICAMP), Brazil under the protocol 52882621.5.0000.5418, and is in accordance with the Helsinki Declaration.

Gingival benign and malignant neoplasms, with a definitive histopathological diagnosis, were selected from the files of the Oral Pathology Laboratory of the Piracicaba Dental School, University of Campinas (Southeast, Brazil); Oral Pathology Laboratory, Federal University of Pernambuco (Northeast, Brazil); Oral Surgical Pathology Laboratory, Federal University of Bahia (Northeast, Brazil); Oral Pathology Laboratory, School of Dentistry, Federal University of Rio de Janeiro (Southeast, Brazil); Oral Pathology Service, State University of Rio de Janeiro (Southeast, Brazil); Oral Pathology Laboratory, School of Dentistry, Federal University of Rio Grande do Sul (South, Brazil); and Oral Laboratory Pathology, João de Barros Barreto University Hospital, Federal University of Pará (North, Brazil), in the period between 1979 and 2020. Only cases with enough clinical information and histopathological diagnosis were evaluated.

This cross-sectional study followed the STROBE statement (15). Clinical and demographic data, such as age, sex, ethnicity, location (maxillary, mandibular, posterior, and anterior gingiva), elementary lesion, the color of mucosal surface, time of complaint, tumor size, type of biopsy performed, clinical diagnoses, and histopathological diagnosis were retrieved from the patient’s records. The histopathological diagnoses were not reviewed. All diagnoses have been established by experienced oral pathologists from hospitals or university institutions with extensive experience in histopathological diagnosis. All subjects regardless of gender or age, who had sufficiently described clinical data in the charts were included in the study. Non-neoplastic lesions, tumors located on the edentulous alveolar ridge, alveolar mucosa, or floor of the mouth, as well as intraosseous lesions that ruptured cortical bone and involved the gingival tissue, were excluded from the sample. In addition, neoplasms were classified as benign or malignant, primary, metastatic or systemic, according to the WHO Classification of Head and Neck Tumors, 2017.

The data collected were analyzed using the SPSS software (SPSS for windows, version 22, SPSS inc, Chicago, IL, USA). The results were assessed with descriptive statistics, with absolute and relative frequencies distribution. The chi-square (X2) test was used to analyze the associations between the evaluated variables. To analyze the differences in the medians between age, tumor size, and time of complaint, the median test of independent samples and the U Mann-Whitney test were used, considering a significance of 5%. To assess the association between clinical and histopathological diagnoses, it was considered that there was an agreement between clinical and histopathological diagnosis in cases in which the final diagnosis was considered in the initial clinical hypothesis. The data were tabulated and expressed in percentages of agreement and non-agreement.

## Results

From 100,026 cases of oral lesions diagnosed in the period of study, 888 (0.9%) were gingival neoplasms. There were 496 (55.9%) males and 391 (44.0%) females, with a mean age of 54.2 years (ranging from 1 to 104 years; SD=20.96). In this series, 264 (29.7%) cases were benign tumors and 624 (70.3%) malignant. Malignant neoplasms were more frequent in older individuals, with the peak of prevalence in people aged 70 years or older (27.4%; *n*=171). For malignant and benign tumors, the most common location was the mandibular and posterior gingiva. The detailed demographic and clinical features of the gingival tumors are described in Table 1 and Table 2.

**Table 1:**
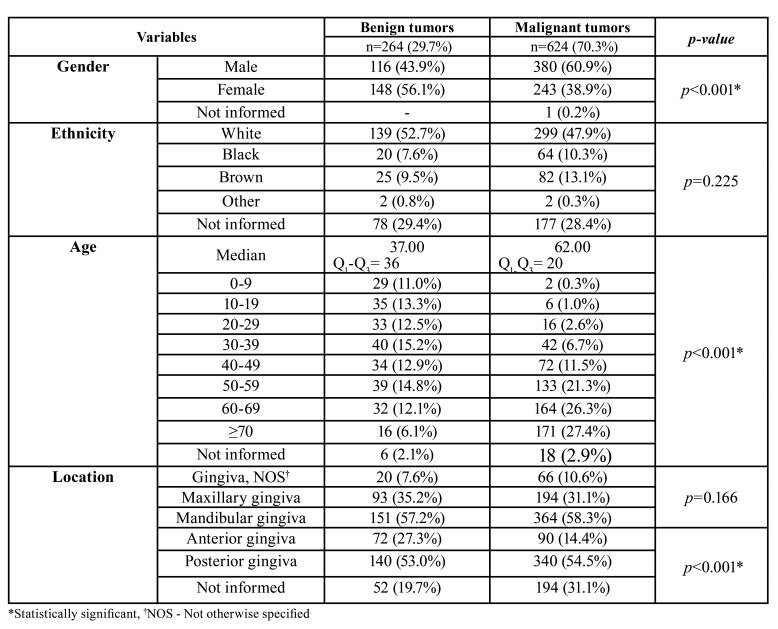
Sociodemographic characteristics and location of gingival neoplasms.

**Table 2:**
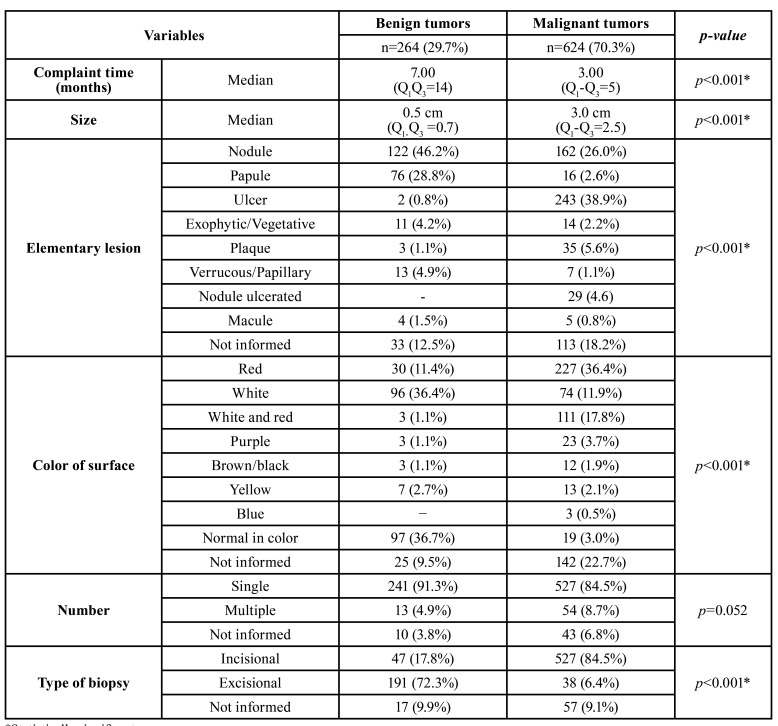
Clinical features of gingival tumors.

The most frequent benign neoplasm was the squamous cell papilloma (65.3%; *n*=174), followed by peripheral odontogenic fibroma (8.0%; *n*=21), neurofibroma (6.1%; *n*=16), lipoma (4.9%; *n*=13), and hemangioma (4.5%; *n*=12). Considering the malignant tumors, the SCC was the most common diagnosed neoplasm (79.1%; *n*=494), followed by non-Hodgkin lymphomas (4.8%; *n*=30). The histological types and the number of each diagnosed tumor are described in Table 3.

**Figure 1:**
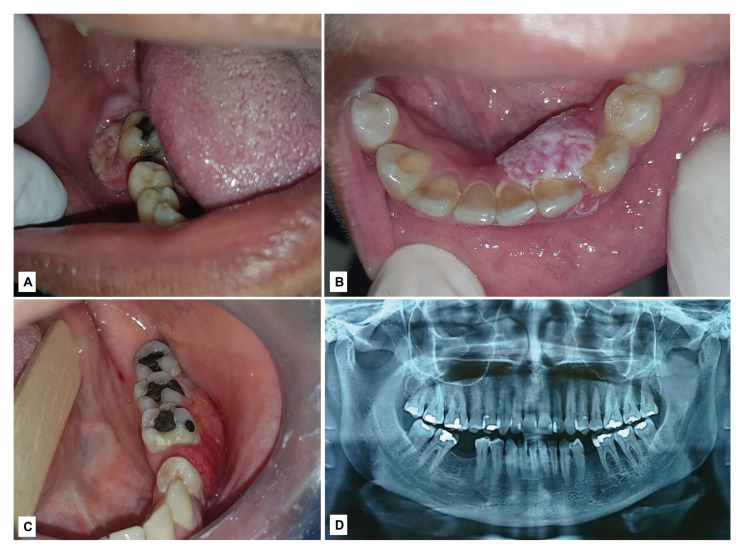
Clinical presentations of gingival squamous cell carcinoma. A - 53-year-old male patient, with an ulcerated lesion with indurated and raised margins in the gingiva of right mandibular second molar. B- 28-year-old female patient with a reddish-white nodule, with papillary surface, involving the buccal and lingual mandibular anterior gingiva at right. C- 42-year-old female patient with a reddish swelling in the left posterior mandibular gingiva, appearing with a granular surface and focal areas of telangiectasia. The first molar presented mobility and the patient was under treatment for periodontitis. D - The same patient of patient seen in C. Panoramic radiograph revealed a mandibular diffuse radiolucency in the region of the left first molar, which caused alveolar bone loss.

The SCC presented mostly as an ulcerated (56.0%, *n*=229) and red (48.8%, *n*=183) lesion, with a size greater than 3 cm in 40.9% (*n*=124) of the cases (Fig. 1). The tumor was more frequent in males (62.3%; *n*=297) than in females (37.7%; *n*=180), and predominantly in individuals aged 70 years or older (32.5%; *n*=152). However, 20 cases (4.0%) occurred in patients younger than 40 years of age. Non-Hodgkin lymphomas, on the other hand, appeared more frequently as a nodule (80%; *n*=20) larger than 2 cm (56.3%; *n*=9), showing a reddish or purple color (69.2%, *n*= 16). The lymphomas were more common in men (76.7%; *n*=23) aged over 50 years (44.8%; *n*=13). Concerning to the metastases, most tumors appeared as a reddish (50.0%; *n*=5) nodule (84.6%; *n*=11) in women (58.8%; *n*=10), aged over 40 years (73.3%; *n*=11). Based on the frequency of clinical features observed in this series, a drew of main clinical profile of the most prevalent malignant tumors is summarized in Table 4.

**Table 3:**
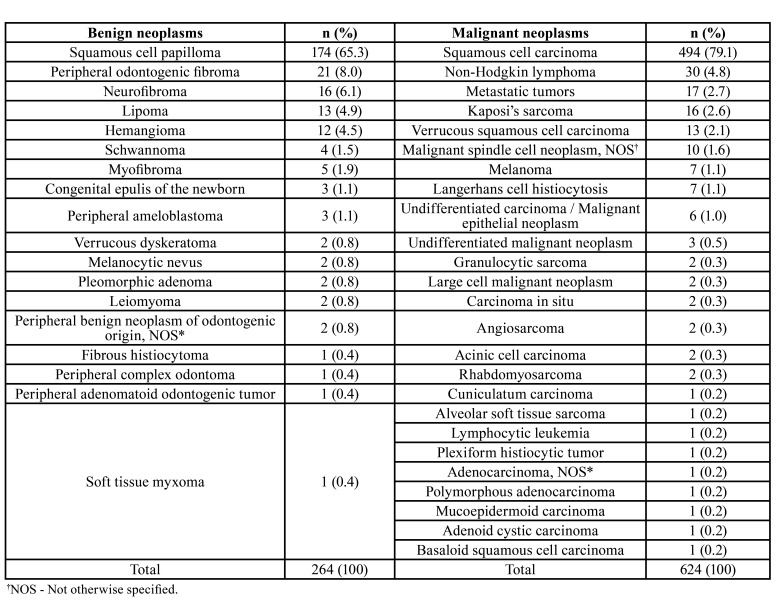
Benign and malignant neoplasms diagnosed in the sample.

**Table 4:**
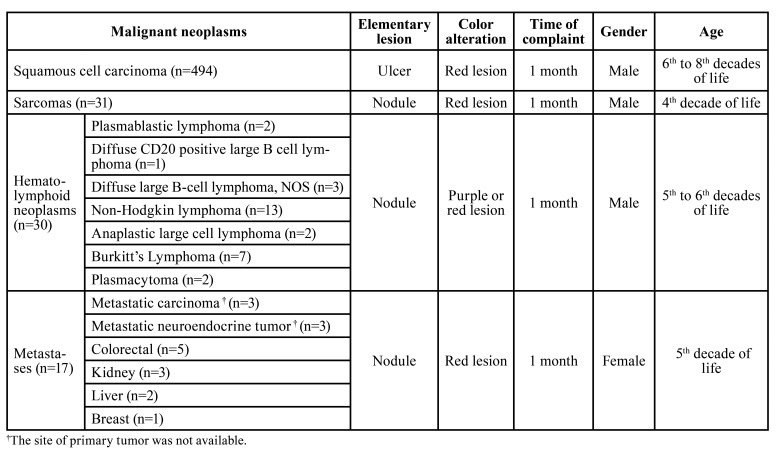
Main clinical profile of most common gingival malignant tumors diagnosed in the sample.

 There was association between the clinical and histopathological diagnoses in 582 (65.5%) cases, but in 246 (27.7%) the clinical diagnosis was not confirmed by histopathological examination. In 60 (6.8%) cases this information was not available. Among the benign tumors, the success rate of the clinical hypothesis was 61.8% (*n*=147), while the percentage of hypotheses that were not confirmed in the histopathological examination was 33.2% (*n*=79). In malignant neoplasms, the association between clinical and histopathological diagnosis was 70.1% (*n*=406), but in 29.9% (*n*=152) of the cases no association was observed. Among the hypotheses listed, reactional lesions, benign and malignant tumors were the most hypothesized diseases. In 69 (11.1%) cases of malignant neoplasms, the lesions were clinically considered to be reactional or of infectious origin.

Statistical analysis revealed that malignant neoplasms were more common in men (*p*<0.001) and the median age was higher in patients with gingiva cancer (*p*<0.001) when compared to the benign tumors, which affected more commonly younger individuals (*p*<0.001). Regarding to the time of duration of the lesions, patients with malignant neoplasms had a lower median time of complaint than those with benign tumors (*p*<0.001). The malignant tumors presented larger size than benign (*p*<0.001), and the incisional biopsy was the most common clinical procedure for diagnosis of malignant neoplasms (*p*<0.001). In contrast, the excisional biopsy was more frequent in benign tumors (*p*<0.001). Furthermore, the cases with clinical diagnoses of malignant lesions were more frequently submitted to incisional biopsy, while hypotheses involving benign lesions were more commonly excised (*p*<0.001). For both malignant and benign neoplasms, the posterior mandibular gingiva was the most common site (*p*<0.001).

## Discussion

The gingiva is an easily accessible site, characterized by mucous tissue with a thin layer of epithelium and connective tissue, which is directly supported on the bone. It represents a relatively uncommon site for the development of neoplasms (6). In this series, after reviewing 100,026 histopathological reports, we identified 888 cases of gingival neoplasms, which corresponds to 0.9% of all histopathological reports retrieved. To the best of our knowledge, this is the second largest series of gingival neoplasms reported in the English-language literature (7). In this study, the gingival neoplasms were more common in males, with a mean age of 53.4 years. It is noteworthy to highlight that the higher prevalence in men is associated with the higher prevalence of malignant tumors in this sample, which occurred significantly more in men. On the other hand, benign neoplasms were more common in women.

Several studies were dedicated to survey all gingival biopsies in a specific population, but none reviewed only gingival neoplasms (2,7,11). These studies showed that most cases consisted of non-neoplastic lesions. A survey of 788 samples revealed that the clinical appearances that most motivated the gingival biopsies were exophytic lesions and/or swellings (45%), changes in the color of the mucosa (39%) and loss of substance (16%) (11). In the present study, nodules, papules, and exophytic lesions also represented the clinical changes most biopsied in the benign neoplasms. In malignant tumors, the most frequent elementary lesion was an ulcer. The most common location was the posterior mandibular gingiva for both benign and malignant tumors (7,13,16). The tumors also appeared as single lesions. The benign neoplasms were more often submitted to an excisional biopsy, whereas the incisional biopsy was commonly performed in malignant tumors, as it is usually preconized by literature (17). Malignant neoplasms were significantly larger than benign tumors. Tumor size influenced the choice of biopsy type. Lesions larger than 3 cm, common in malignant tumors, were more frequently submitted to incisional biopsy (18,19). The clinical diagnosis also influenced the choice of the clinical procedure for diagnosis. Incisional and excisional biopsies were more common in malignant and benign neoplasms, respectively, as recommended by literature (20).

The benign neoplasms often showed whitish or normal coloration of the lesion surface, probably because most cases were squamous cell papilloma. Li *et al*. (7) also found the squamous cell papilloma as the most common benign tumor occurring on gingiva. This lesion usually appears as a whitish papule or nodule, with papillary and verrucous surface (17,21,22). The most prevalent benign tumor in our sample after squamous papilloma was hemangioma, lipoma, schwannoma, and myofibroma.

The SCC was the most common malignant gingival neoplasm, similar to found in other series (2,7,11). In our study, SCC commonly appeared as a gingival ulcerated lesion in men aged over 50 years. Another series found similar clinical features, but in patients with a mean age of 41 years (16). In contrast, Fitzpatrick *et al*. (13) reported exophytic and verrucous lesions as the most often clinical presentation for gingival SCC in patients with a lower mean age. Most patients developed a SCC in the mandibular gingiva, similar to our findings (13,16). Regarding the risk factors, gingival SCC seems to be less associated with tobacco and alcohol consumption when compared to other oral sites, mainly tongue and floor of the mouth. A survey showed that SCC of oral tongue is 38 times more likely to be diagnosed in smokers when compared to gingival tumors (23). In addition, another study revealed that the percentage of smokers is relatively lower for gingival SCC when compared to SCC of the tongue and floor of the mouth (24). Another survey found that 52.5% of patients with gingival SCC were non-smokers (18). In the current series, information on habits and risk factors was not available.

Other malignant tumors can occur in the gingival tissue, such as leukemic infiltrations, lymphomas, sarcomas, Langerhans cell histiocytosis, melanomas, and metastases (25), as observed in the present study. Kaposi's sarcoma (KS) and plasmablastic lymphoma (PBL) are malignant neoplasms that frequently occur in immunocompromised patients, particularly HIV-associated immunosuppression (26). KS is a vascular neoplasm of endothelial origin that affects mucocutaneous tissues, caused by human herpesvirus (HHV-8) infection. Oral manifestations of KS occur in all variants, but it is predominantly seen in HIV-infected individuals (27). In the present study, four patients had HIV infection at the time of diagnosis of neoplasia. The cases were treated with antiretroviral therapy combined with chemotherapy. The PBL is an uncommon and aggressive form of diffuse large B-cell lymphoma characterized by the proliferation of immunoblasts and plasmablasts, strongly associated with EBV infection. Most cases occur in immunocompromised patients associated with HIV infection (28). PBL was initially described in oral cavity and may be the first sign of HIV-infection (29). In this series, one patient was identified with HIV-infection after the diagnosis of PBL. Although the emergence of antiretroviral therapy has decreased considerably the incidence of neoplasms linked to AIDS, when faced with these neoplasms, particularly PBL and KS, the possibility of HIV infection should be investigated (26).

Overall, metastasis of malignant tumors to the oral cavity is rare and usually indicates the possibility of disseminated disease linked to a worse prognosis (9,30). In our study, 17 cases of gingival metastases were described. A recent systematic review revealed that the gingiva is the most common site for oral soft tissue metastases (10). Based on the evidence that metastasis is a highly regulated and specific process, some authors hypothesize that some local factors in the gingival tissue, such as chronic inflammation, could favor the attraction of circulating tumor cells to the gingiva (9,10). In addition, there is evidence of an association between the presence of teeth and the occurrence of gingival metastasis, because 80% of the cases occurred in dentate patients. Only one-third of edentulous patients with oral metastases presented gingival lesions. In these patients, the metastases were located in other sites of the oral cavity, such as the tongue (9). For Hirshberg *et al*. (30), malignant cells can be attracted through the extensive network of capillaries that form the chronic gingival inflammation. This microenvironment, present in chronically inflamed gingiva, can favor the progression of metastatic cells, since in the past, the chronic inflammation has been associated with tumorigenesis processes, such as cell transformation, promotion, survival, proliferation, and invasion, as well as angiogenesis and metastasis (10,30).

Regarding the clinical diagnoses listed by the clinicians, lesions of different etiologies were hypothesized. Some non-neoplastic lesions that constitute the differential diagnosis of gingival tumors, such as pyogenic granuloma, peripheral giant cell granuloma, peripheral ossifying fibroma, and infectious diseases, were considered, as observed in other published series (11,13). In the present study, although 65.5% of the cases had the clinical diagnosis confirmed after histopathological analysis, no association between the clinical and microscopic diagnoses was observed in 27.7% of the cases. The differential diagnosis of gingival tumors is challenging because the clinical presentation often mimic indolent and non-neoplastic lesions (4,5). Therefore, in cases of persistent lesions that do not heal after plaque removal or after periodontal standard therapy is instituted, the clinician should perform a biopsy and send the specimen for histopathological analysis.

The present study has several strengths, including the second-largest series of gingival neoplasms already reported, which discusses the clinical features and differential diagnosis. However, some limitations need to be considered, mainly because it represents a retrospective study that evaluated lesions located on a limiting site, as the gingival tissue. The gingiva is a scarce tissue when compared to other oral sites. For this reason, although the study had rigorous inclusion criteria, some cases included in this study could be sited on the edentulous alveolar ridge because the location recorded may have been incorrect. On the contrary, cases of gingival neoplasms could have been missed. The tumors tend to grow and invade contiguous anatomical sites, such as floor of the mouth, palate, and the buccal mucous fold. This fact could also lead to an incorrect recording of the exact location of the lesion.

In conclusion, malignant gingival tumors are notably more frequent than benign, and usually appear as an ulcer or a reddish/purple nodule, with large dimensions, and in older individuals. Nevertheless, gingival cancers also occur in young patients, including SCC. Malignant neoplasms should be considered in the differential diagnosis of a gingival ulcerated lesion or a red/purple nodule with short-term growth, without evidence of healing. In these cases, an incisional biopsy must be performed to prompt diagnosis and treatment.
